# FOP Is a Centriolar Satellite Protein Involved in Ciliogenesis

**DOI:** 10.1371/journal.pone.0058589

**Published:** 2013-03-12

**Authors:** Joanna Y. Lee, Tim Stearns

**Affiliations:** 1 Department of Biology, Stanford University, Stanford, California, United States of America; 2 Department of Genetics, Stanford School of Medicine, Stanford, California, United States of America; The Hong Kong University of Science and Technology, Hong Kong

## Abstract

Centriolar satellites are proteinaceous granules that are often clustered around the centrosome. Although centriolar satellites have been implicated in protein trafficking in relation to the centrosome and cilium, the details of their function and composition remain unknown. FOP (FGFR1 Oncogene Partner) is a known centrosome protein with homology to the centriolar satellite proteins FOR20 and OFD1. We find that FOP partially co-localizes with the satellite component PCM1 in a cell cycle-dependent manner, similarly to the satellite and cilium component BBS4. As for BBS4, FOP localization to satellites is cell cycle dependent, with few satellites labeled in G_1_, when FOP protein levels are lowest, and most labeled in G_2_. FOP-FGFR1, an oncogenic fusion that causes a form of leukemia called myeloproliferative neoplasm, also localizes to centriolar satellites where it increases tyrosine phosphorylation. Depletion of FOP strongly inhibits primary cilium formation in human RPE-1 cells. These results suggest that FOP is a centriolar satellite cargo protein and, as for several other satellite-associated proteins, is involved in ciliogenesis. Localization of the FOP-FGFR1 fusion kinase to centriolar satellites may be relevant to myeloproliferative neoplasm disease progression.

## Introduction

The centrosome is the main microtubule-organizing center of animal cells. Each centrosome consists of two centrioles associated with pericentriolar material that nucleates and organizes microtubules. Microtubules nucleated from the centrosome function in mitosis, vesicular trafficking, cell motility, and determining cell shape. In addition to these structures, many cells also have an array of granules 70–100 nm in diameter, known as centriolar satellites, that localize around the centrosome in a microtubule-dependent fashion [1], [2], [3].

During cell division, the centrosome duplicates such that each daughter cell inherits a centriole pair. Each existing centriole templates the growth of a new centriole, resulting in an old and new centriole within each pair. The older centriole, referred to as the mother centriole, bears appendages that are required for the formation of the primary cilium, a sensory organelle that serves important roles in signaling [4], [5], [6], [7], [8]. Mutations in genes required for primary cilium function are responsible for several human diseases, termed ciliopathies. Ciliopathies share a set of phenotypes, including cystic kidneys, retinal degeneration, obesity, diabetes, and neurocognitive deficits [9], [10]. Interestingly, mutations in genes encoding centriolar satellite proteins are responsible for some cases of the ciliopathies Bardet-Biedl, Joubert, and oral-facial-digital syndromes [10], [11], [12].

PCM1 is thought to be the core component of centriolar satellites and its depletion causes loss of satellite structures [3], [11], [13], [14], [15], [16]. Disruption of satellites by PCM1 depletion mislocalizes centrosome components including ninein and pericentrin [17]. Recent studies show that the satellite proteins Cep72 and Cep290 are required for the proper transition of BBS4 from satellites to the primary cilium during ciliogenesis [18]. This suggests that satellites might serve as a platform for the regulated recruitment and release of ciliary proteins. Although there are about a dozen proteins known to localize to centriolar satellites [2], little is known about the details of their organization or their full composition.

FOP (FGFR1 oncogene partner) was first described as the fusion partner of FGFR1 in a leukemia-associated chromosomal translocation [19]. FOP was identified as a putative centrosome protein in a mass spectrometry proteomic study of centrosomes [20]. Further evidence linking FOP to the centrosome came from a study identifying an interaction between FOP and the centrosome protein CAP350 [21]; FOP localized to the centrosome throughout the cell cycle, with that localization dependent on interaction with CAP350. Furthermore, short-term depletion of FOP by siRNA resulted in microtubule anchoring defects and loss of centrosomal EB1, a microtubule plus-end-associated protein. Deletion of the gene encoding FOP in DT40 chicken cells resulted in G_1_ arrest followed by apoptosis [22].

FOP shares homology with reported satellite proteins FOR20 (FOP-related protein of 20 kD) and OFD1 [11], [16]. FOP, FOR20, and OFD1 each have an N-terminal Lis1 homology (LisH) domain although they are otherwise dissimilar [16]. Both FOR20 and OFD1 localize to the centrosome and centriolar satellites [23]. Recently it has been shown that FOR20 may contribute to ciliogenesis through a role in transition zone assembly in Paramecium [23]. Depletion of either FOR20 or OFD1 blocks ciliogenesis and alters the distribution of PCM1 [24]. Here we show that FOP is also a component of centriolar satellites, co-localizing with PCM1 in a cell cycle-dependent manner, and that depletion of FOP disrupts ciliogenesis.

## Results

### FOP Localizes to Centrioles and Centriolar Satellites

**Figure 1:**
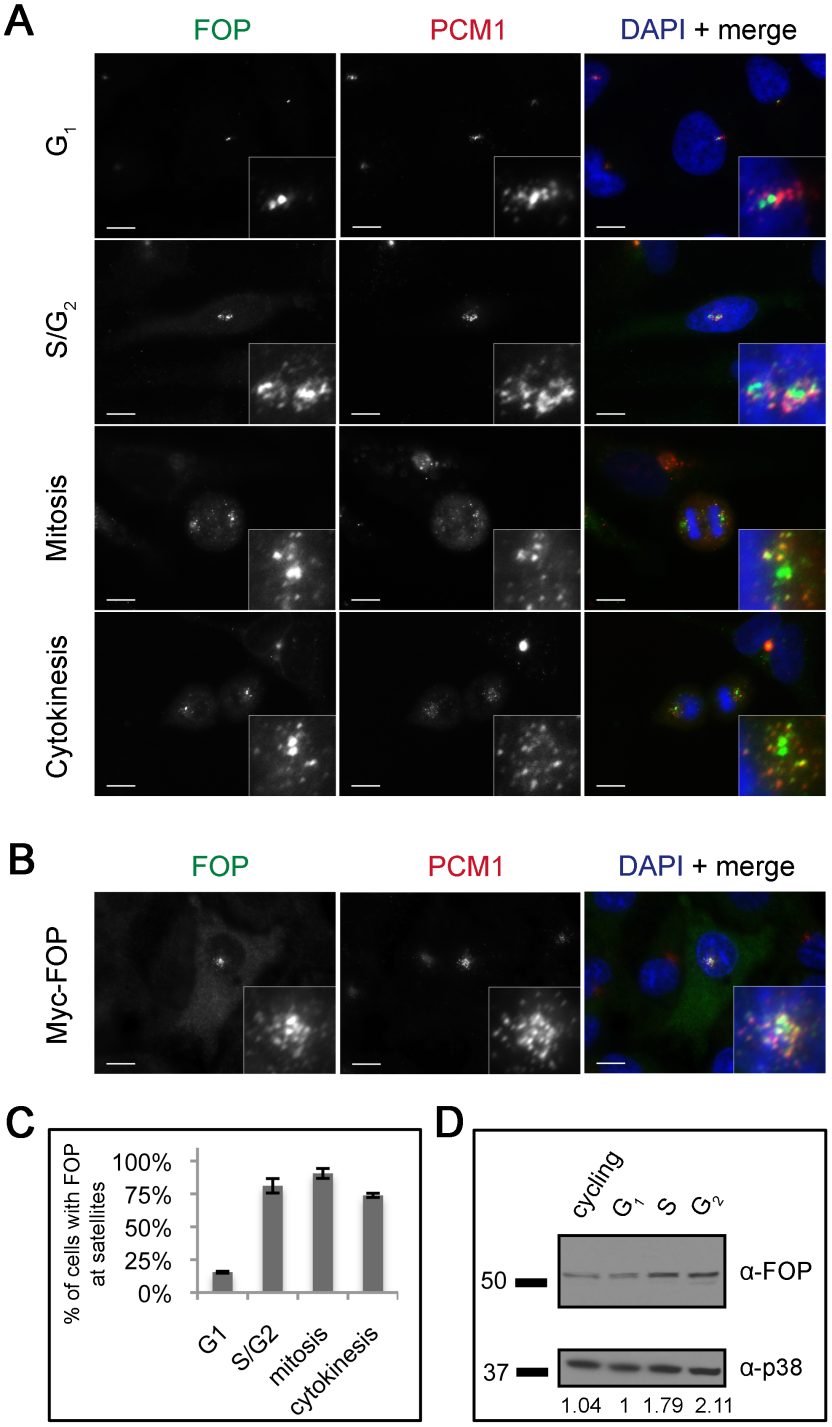
FOP localizes to centrioles and centriolar satellites in a cell cycle-dependent manner. (**A**) Asynchronous HeLa cells stained with antibodies against FOP (green) and PCM-1 (red) showing FOP localization at different points in the cell cycle. (**B**) RPE-1 cells transfected with Myc-FOP and stained with antibodies against Myc (green) and PCM-1 (red). DNA is stained using DAPI (blue). Scale bars: 10 µm; insets: 5× magnification. (**C**) Quantification of percent of cells with FOP satellite localization during different points in the cell cycle. Bars are mean ± std. dev. from two experiments. Total N  = 200, 42, 125, 42, for G_1_, S/G_2_, mitosis, and cytokinesis, resp. (**D**) Western blot analysis of endogenous FOP protein levels in different stages of the cell cycle. Lysates from asynchronous or synchronized HeLa cells were probed with antibodies against FOP and p38 as a loading control. Relative FOP protein levels are calculated as the ratio of FOP/p38 for each lane, normalizing the G_1_ level to 1.

Previously, FOP has been shown to co-localize with γ-tubulin at centrosomes [21]. Given the relatedness of FOP to OFD1 and FOR20, both of which localize to centriolar satellites and other centrosome structures, we investigated the localization of FOP in more detail. HeLa cells were stained with antibodies against FOP and the centriolar satellite protein PCM1 (Fig. 1A). The G_1_ and G_2_ phases of the cell cycle were distinguished by the distinct PCM1 staining pattern and number of FOP centriolar foci, which corresponds to centriole number by centrin staining (Fig. S1). FOP localized to centrioles at all cell cycle stages, apparent as two foci within each centrosome. In addition, FOP colocalized with PCM1 at a subset of centriolar satellites (Fig. 1A). We found that Myc-tagged FOP expressed by transient transfection in RPE-1 cells localized to centrioles and co-localized with PCM1 at centriolar satellites similarly to the endogenous protein (Fig. 1B). Endogenous FOP localized to satellites in all cell cycle stages except G_1_, in which its localization was limited to the centrioles (Fig. 1C). Depletion of FOP reactivity by incubation of the antibody with purified recombinant FOP resulted in loss of staining of both centrioles and satellites, demonstrating specificity of the antibody (Fig. S2).

Transiently expressed Myc-FOP protein often localized to satellites in G_1_ cells, unlike the endogenous protein (Fig. 1B). This suggested that expression of Myc-FOP above the endogenous level of FOP in G_1_ resulted in localization to satellites, raising the possibility that the absence of endogenous FOP from satellites in G_1_ cells might be due to cell cycle-dependent changes in protein level. To test this, we determined the amount of endogenous FOP protein in HeLa cells synchronized in different phases of the cell cycle. Relative to G_1_ cells, cells arrested in S and G_2_ had a 1.79-fold and 2.11-fold increase in FOP protein level, respectively (Fig. 1D). Our data suggest that FOP satellite localization is correlated with FOP protein levels, however additional levels of regulation cannot be discounted.

**Figure 2:**
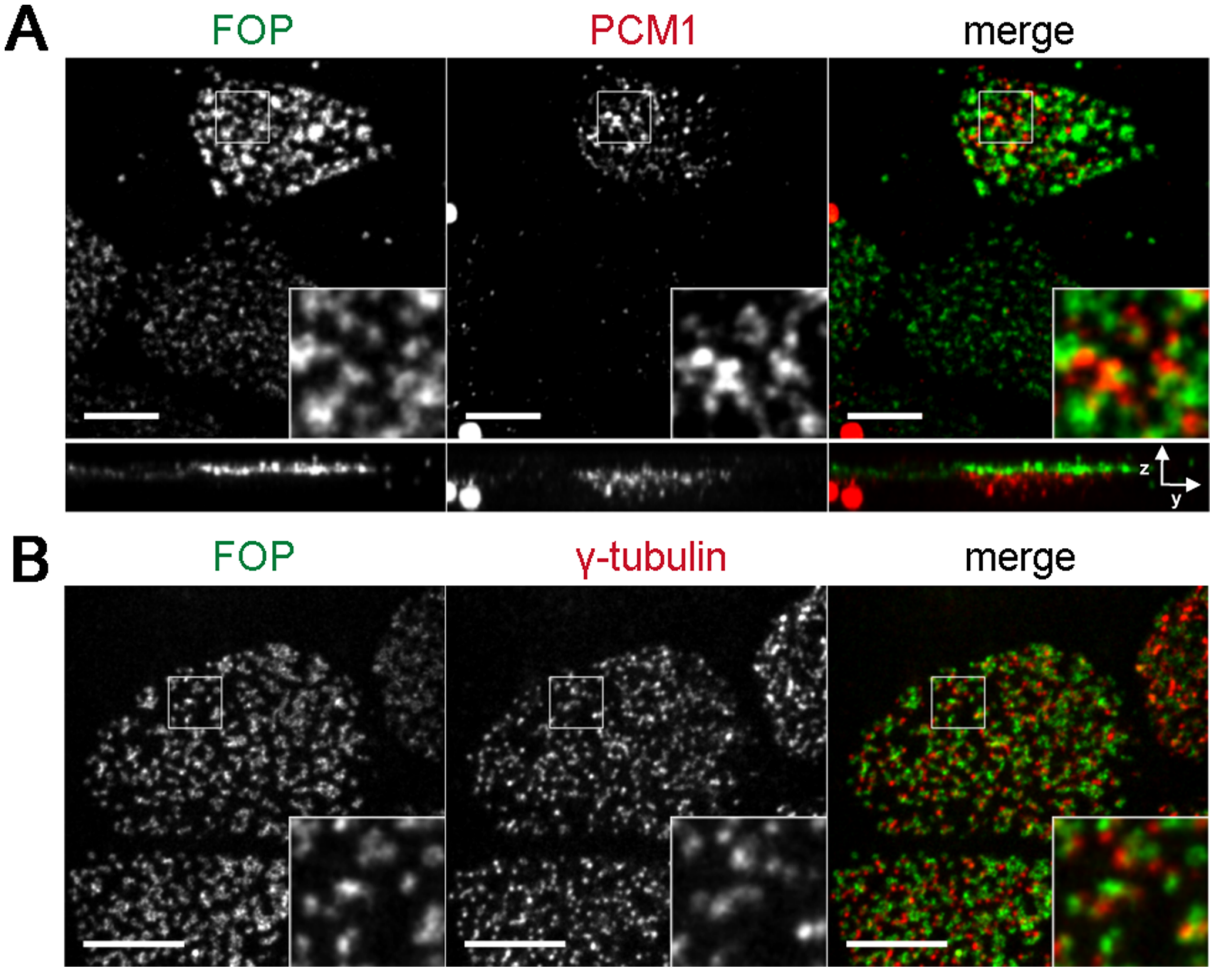
FOP localizes to the basal body layer of multiciliated cells. Mouse tracheal epithelial cells grown on filters and induced to differentiate by establishment of air-liquid interface. Cells were fixed in paraformaldehyde and stained with antibodies against FOP (green) and PCM-1 (red) to mark satellites (**A**) or γ-tubulin (red) to mark basal bodies of multiciliated (**B**). Images shown are maximum projections. Scale bars: 5 and 6 µm resp.; insets: 3× magnification.

We also examined the localization of FOP in multiciliated tracheal epithelial cells, a cell type that forms hundreds of centrioles during differentiation and has a distinctive distribution of PCM1 early in the process of centriole formation [25]. PCM1 localizes to the vicinity of immature centrioles in cells undergoing differentiation, but is almost completely absent in mature multiciliated cells [25]. FOP localized apical to PCM1 in differentiating multi-ciliated cells, consistent with FOP associating with the nascent centrioles (Fig. 2A). In mature cells, FOP localized to basal bodies, but did not precisely co-localize with the γ-tubulin foci that define part of the basal body layer (Fig. 2B), instead localizing to puncta adjacent to γ-tubulin, possibly representing the centrioles themselves, consistent with the localization in cycling cells.

### FOP is Recruited to BBS4-GFP Satellites and Aggregates, but not Cilia

**Figure 3:**
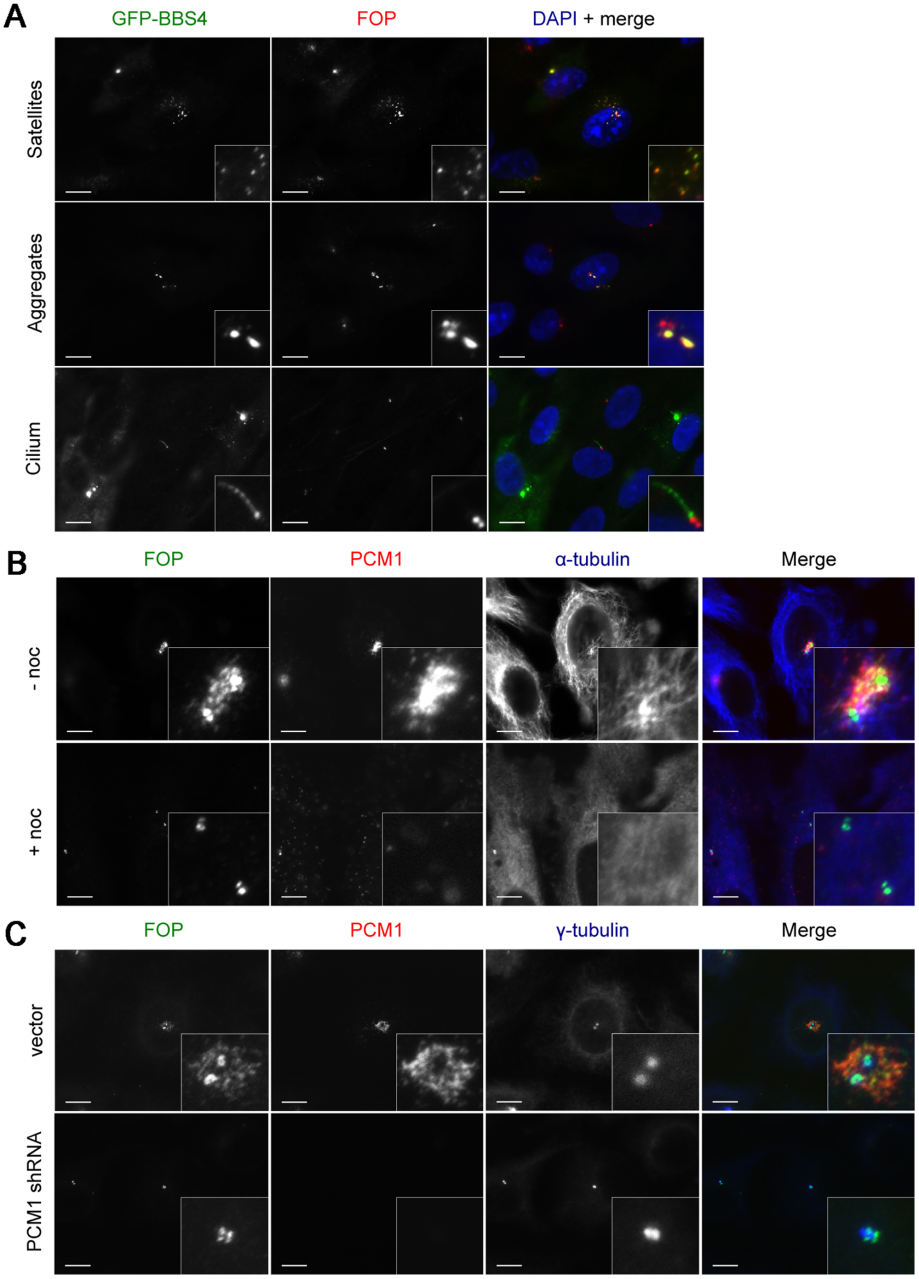
Factors affecting FOP localization to centriolar satellites. (**A**) Asynchronous ^LAP^BBS4-hTERT-RPE1 cells stained with antibodies against GFP (green) and FOP (red) showing FOP localization to BBS4-containing satellites and protein aggregates. DNA is stained using DAPI (blue). Serum starved ^LAP^BBS4-hTERT-RPE1 cells showing localization of ^LAP^BBS4 labeled by antibodies against GFP (green) and FOP (red) in the presence of a primary cilium. (**B**) HeLa cells released from thymidine-arrest to enrich for G_2_ cells and treated with DMSO alone or 10 µg/ml nocodazole. Following treatment, cells were fixed and stained with antibodies against FOP (green), PCM-1 (red), and α-tubulin (blue). (**C**) HeLa cells transfected with PCM-1 shRNA or vector alone and released from thymidine-arrest to enrich for G_2_ cells. Cells were fixed and stained with antibodies against FOP (green), PCM-1 (red), and γ-tubulin (blue). Scale bars: 10 µm; insets: 5× magnification.

Like FOP, the BBS4 protein localizes to satellites during S, G_2_ and M phases, but is absent from satellites in G_1_ [14]. To compare FOP and BBS4 satellite localization, ^LAP^BBS4-hTERT-RPE1 cells [26] were stained for GFP, to localize BBS4, and FOP (Fig. 3A). FOP colocalized with GFP-BBS4 to satellites in non-G_1_ cells. FOP colocalized almost completely with ^LAP^BBS4, but less so with PCM1 (Fig. 1A & Fig. 3A) indicating that FOP and ^LAP^BBS4 are similarly distributed among satellites. In G_1_ cells, both proteins were lost from satellites; ^LAP^BBS4 relocalized to the primary cilium, and FOP remained localized to centrioles. We never observed localization of FOP to the primary cilium, even in FOP overexpressing cells (Fig. 3A). Interestingly, some ^LAP^BBS4-hTERT-RPE1 cells form ^LAP^BBS4 aggregates (Fig. 3A). These aggregates also contain FOP, suggesting that ^LAP^BBS4 aggregates recruit endogenous FOP, similar to the recruitment of other satellite proteins by BBS4 [14], [16].

### FOP Satellite Localization is Dependent on Microtubules and PCM1

The punctate pericentrosomal distribution of known centriolar satellite proteins is dependent both on the presence of PCM1 protein and a centrosomally-focused interphase microtubule array [11], [13], [14], [16]. We tested the dependence of FOP localization on these factors. First, the localization of FOP and PCM1 was assessed in HeLa cells in which microtubules were depolymerized by treatment with nocodazole. FOP and PCM1 dispersed upon depolymerization of microtubules (Fig. 3B), and some of the dispersed puncta, presumably representing dispersed satellites, co-stained for both PCM1 and FOP. In contrast, the localization of FOP to centrioles was unaffected by microtubule depolymerization (Fig. 3B). Depletion of PCM1 caused loss of PCM1-staining satellites. PCM1 depletion also caused loss of FOP satellite localization, but had no effect on FOP staining at centrioles (Fig. 3C). Thus the FOP-containing foci are canonical centriolar satellites, and FOP localizes to centrioles independent of those satellites.

### FOP is Required for Ciliogenesis in RPE-1 Cells

**Figure 4:**
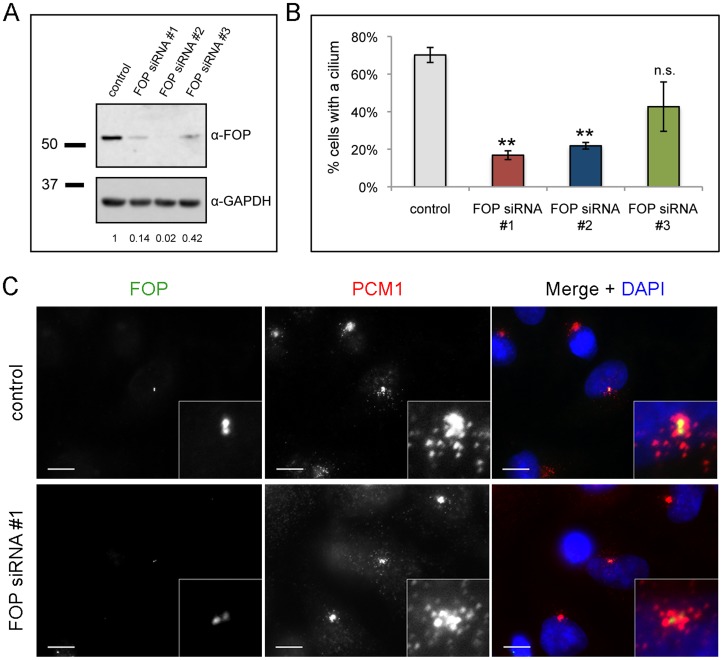
FOP depletion reduces frequency of ciliated cells. (**A**) Western blot analysis of lysates from RPE-1 cells transfected with nontargeting (control) or FOP siRNA #1, #2, or #3. Relative FOP protein levels are calculated as the ratio of FOP/GAPDH for each lane, normalizing the control level to 1. (**B**) Quantification of percent of cells with cilia following transfection with control or one of three FOP siRNAs and serum starvation. Bars are mean ± SEM from three experiments, N  = 200 cells per category per experiment. **p<0.05, n.s. not significant compared to control. (**C**) HeLa cells transfected with control or FOP siRNA, fixed, and stained with antibodies against FOP (green) and PCM-1 (red). DNA is stained using DAPI (blue). Scale bars: 10 µm; insets: 5× magnification.

Many proteins associated with satellites are involved in formation and function of the primary cilium [11], [13], [16], [26]. We tested whether FOP depletion affects cilium formation. Efficient depletion of FOP by transfection with an siRNA targeting FOP has been reported [21]; using an siRNA of the same sequence (FOP siRNA #1) and two additional siRNAs targeting unrelated sequences (FOP siRNA #2 and #3), we achieved 86%, 98%, and 58% depletion of FOP in RPE-1 cells, respectively, compared to transfection with nontargeting siRNA (Fig. 4A). To test whether FOP depletion results in a defect in primary cilium formation, we transfected RPE-1 cells with FOP siRNAs #1–3, and 72 h following transfection assayed by immunofluorescence for presence of a cilium. FOP siRNA #1 and #2 produced similar, statistically significant decreases in ciliogenesis (76.0% and 68.9%, respectively), whereas FOP siRNA #3 did not (39.1%) (Fig. 4B), consistent with its reduced efficacy of depletion. The amount of FOP at centrioles was not appreciably altered in depleted cells using any of the three siRNAs, although the depletion did eliminate the satellite labeling normally present in the fraction of G_1_ cells (Fig. 4C) and G_2_ cells (Fig. S3). Presumably this reflects higher affinity association of the FOP with the centrioles than other sites, and a substantial satellite/cytoplasmic pool of FOP under normal conditions, and is similar to our experience with other centriole proteins (data not shown). As for the satellite proteins Cep72, BBS4, OFD1, and FOR20 [11], [14], [16], [18], depletion of FOP did not affect the presence of PCM1-positive satellites. In cells depleted of FOP, the satellites had a distribution similar to that in control cells (Fig. 4C); this is in contrast to the effect of depletion of some other satellite proteins in which the satellites become either dispersed or more tightly clustered around the centrosome.

**Figure 5:**
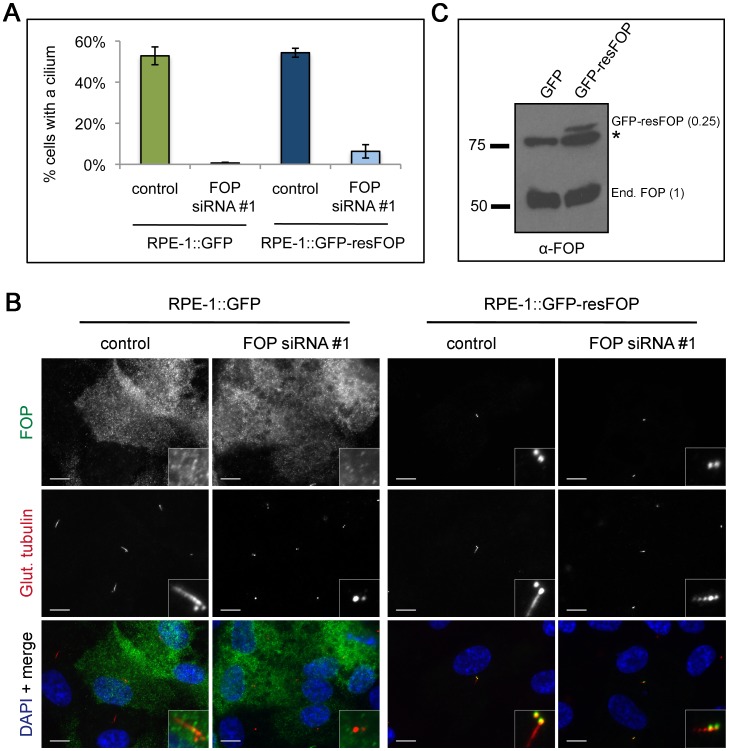
FOP depletion is weakly rescued by expression of siRNA resistant FOP. (**A**) Quantification of stably expressing GFP or GFP-resFOP cells with cilia following transfection with control or FOP siRNA #1 and serum starvation. Bars are mean ± SEM from three experiments, N  = 200 cells per category per experiment. (**B**) Western blot analysis of lysates from RPE-1:GFP or RPE-1::GFP-resFOP cells probing for relative levels of endogenous (End.) FOP and GFP-resFOP. * marks non-specific band. Relative GFP-resFOP protein level, calculated as the ratio of GFP-resFOP/End. FOP for RPE-1::GFP-resFOP, is equal to 0.25. (**C**) RPE-1:GFP or RPE-1::GFP-resFOP cells transfected with control or FOP siRNA, serum starved for 24 hours, fixed, and stained with antibodies against FOP (green) and glutamylated-tubulin (red). DNA is stained using DAPI (blue). Scale bars: 10 µm; insets: 5x magnification.

As an additional control for the specificity of the depletion ciliogenesis phenotype, we tested the ability of an RNAi-resistant mutant of FOP to rescue the phenotype. RPE-1 cells stably expressing GFP-tagged siRNA #1-resistant FOP (GFP-resFOP) or GFP alone were transfected with FOP siRNA #1 or control siRNA. 72 h following transfection, cells were assayed by immunofluorescence for presence of a cilium. 52.8% of RPE-1::GFP control cells, but only 0.7% of FOP depleted cells, formed a cilium (Fig. 5A & B). Complementation of the FOP depletion phenotype by transfection of FOP siRNA in RPE-1::GFP-resFOP cells resulted in a 9-fold increase (6.3%) in FOP siRNA-treated cells forming a cilium. Although the fraction of cells rescued by expression of the RNAi-resistant FOP construct is low, we note that comparison of RPE-1::GFP and RPE-1::GFP-resFOP cell lysates (Fig. 5C) showed that the level of GFP-resFOP is four times lower than endogenous FOP. In sum, these results show a dose-dependent requirement for FOP in making a primary cilium.

### Myc-FOP-FGFR1 localizes to centriolar satellites

**Figure 6:**
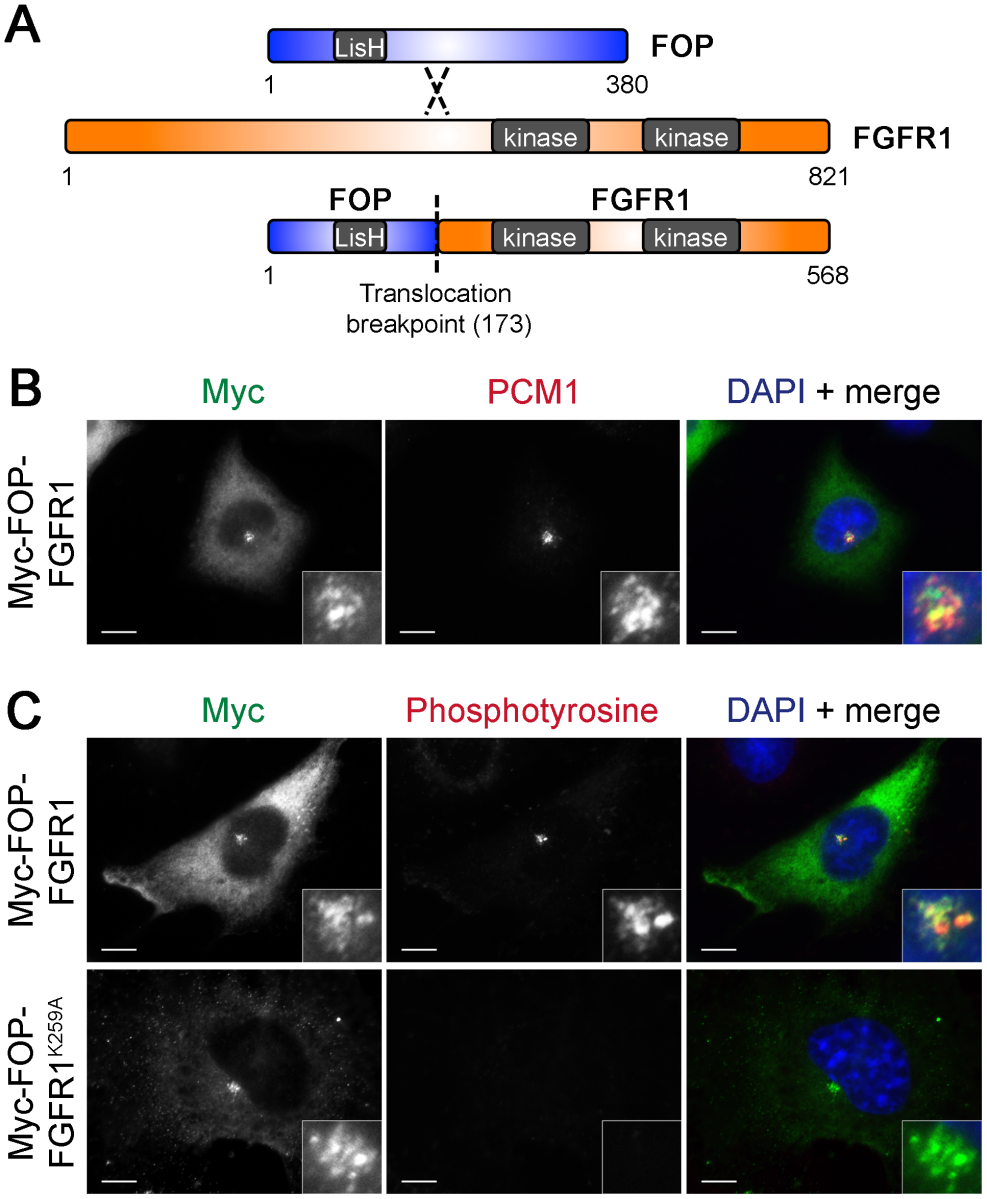
Myc-FOP-FGFR1 localizes to centriolar satellites. (**A**) Schematic of domain structure of FOP, FGFR1, and the FOP-FGFR1 fusion showing FOP LisH domain, FGFR1 kinase domains, and the translocation breakpoint. (**A**) RPE-1 cells transfected with Myc-FOP-FGFR1 and stained with antibodies against Myc (green) and PCM-1 (red) or phosphotyrosine (red) (**B**). DNA is stained using DAPI (blue). Scale bars: 10 µm; insets: 5× magnification.

FOP-FGFR1, a fusion protein joining the N-terminus of FOP and kinase domain-containing C-terminus of FGFR1 (Fig. 6A), causes myeloproliferative neoplasm (MPN), a form of leukemia. FOP-FGFR1 has been shown to localize to the centrosome, where it increases the amount of phosphotyrosine [27]. Interestingly, PCM1 and JAK2 form another fusion pair known to cause MPN [28], [29]. As PCM1 and FOP are both found in MPN fusions and both localize to centriolar satellites, it is possible that localization of the active kinase fragments to centriolar satellites may be one mechanism for MPN. To determine if FOP-FGFR1 localizes to satellites in addition to its known centrosome localization, Myc-tagged FOP-FGFR1 was expressed in RPE-1 cells. Like the FOP protein, Myc-FOP-FGFR1 localized to centrioles, and co-localized with PCM1 at a subset of centriolar satellites (Fig. 6B). Thus, the N-terminal fragment of FOP retained in the FOP-FGFR1 fusion (1–173), which contains the LisH domain, is sufficient to target the fusion to satellites as well as the centrosome, as previously reported [30].

We tested whether FOP-FGFR1 localization to satellites causes an increase in phosphotyrosine at satellites, as it does at the centrosome. RPE-1 cells were transfected with Myc-tagged FOP-FGFR1 and labeled with anti-phosphotyrosine antibody. Myc-FOP-FGFR1 expression caused an increase in phosphotyrosine staining at satellites in addition to the centrosome, which did not occur in cells transfected with the kinase-dead mutant Myc-FOP-FGFR1^K259A^ (Fig. 6C) or FGFR1 targeted to other subcellular locations (data not shown).

## Discussion

FOP was initially identified as a fusion with the FGFR1 gene in a case of myeloproliferative neoplasm [19], and was subsequently shown to localize to the centrosome and be involved in centrosome functions [21]. The results presented here extend the previous work by showing that FOP is, in addition, associated with centriolar satellites during part of the cell cycle and is required for ciliogenesis.

The association of FOP with centriolar satellites is inversely correlated with ciliogenesis in cycling cells that make a primary cilium. Most cells make a cilium in G_1_, and FOP is absent from satellites during G1; the cilium is usually lost at some point after the G_1_/S transition, when FOP is associated with satellites. FOP is present on centrioles throughout the cell cycle. Stowe et al. (2012) have proposed that for some proteins that associate with centriolar satellites, that association serves to restrict their localization, such that when bound to satellites they are prevented from localizing to the centrosome or cilium. A simple model would be that satellites in G_2_ cells perform a similar role for FOP; when cilia are resorbed, satellites sequester the excess FOP protein that is no longer needed in ciliogenesis. We note that the requirement for FOP in efficient primary cilium formation is in contrast with a previous study investigating the phenotype of FOP depletion [31], which observed no ciliogenesis phenotype. However, that study only examined FOP depletion in the context of a high-throughput screen of centrosome proteins for involvement in ciliogenesis and did not correlate depletion of FOP protein with cilium presence.

The transient, cell-cycle dependent localization of FOP at satellites suggests that it is regulated in some way by association with centriolar satellites rather than being a core component. Such a protein could be considered to be a “cargo” of the satellites. This is supported by our results that FOP depletion did not affect satellite number or localization. BBS4 and FOP have a similar cell cycle-regulated pattern of satellite localization and they colocalize to the same subset of satellites. Stowe, et al. showed that BBS4 is released from satellites in a Cep72/Cep290-dependent manner [18]; it is possible that this module also regulates FOP localization. BBS4 differs from FOP in that both overexpression and depletion of BBS4 alter PCM1 localization [14], neither of which is the case for FOP.

FOP is only one of a number of centrosome proteins that are found fused to tyrosine kinases in myeloproliferative neoplasms [32]. The functional significance of centrosome-kinase fusions in myeloproliferative neoplasms is not well understood, however it has been hypothesized that aberrant kinase localization is a factor in the disease phenotype [32], [33]. We have shown that FOP-FGFR1 localizes to satellites and that this results in accumulation of tyrosine phosphate at satellites, raising the possibility that interfering with satellite function by aberrant phosphorylation of satellite proteins contributes to the disease phenotype. It will be interesting to test whether FOP-FGFR1 activity at satellites plays a part in the aberrant proliferation of cells observed in myeloproliferative neoplasm patients.

## Materials and Methods

### Plasmids

cDNAs for human FOP (GenBank: BC011902.2) and FGFR1 (GenBank: BC015035.1) were obtained from Open Biosystems. Full-length FOP was PCR-amplified, the FOP-FGFR1 fusion was generated using precise gene fusion by PCR [34], and the FOP-FGFR1^ K259A^ mutant generated by site-directed mutagenesis. An siRNA resistant FOP (resFOP) clone was generated by making three consecutive synonymous base pair changes in the center of the siRNA targeted region using overlapping PCR with the following primers: 5′-tagaagtgatcagAcgTtgCcaacagaaag-3′ and 3′-ctttctgttgGcaAcgTctgatcacttcta-5′. PCR products were cloned into pDONR221 using the Invitrogen Gateway system. Subsequent Gateway recombination reactions using pCS2+6xMyc DEST provided by M. Nachury (Stanford University, Stanford, CA) and pcDNA-DEST47 (Invitrogen) were used to produce Myc-FOP (pTS2321), Myc-FOP-FGFR1 (pTS2305), Myc- FOP-FGFR1^K259A^ (pTS2505), and GFP-resFOP (pTS2896).

### Antibodies

Monoclonal anti-FOP (Abnova) antibodies were used at 1∶1000 for immunofluorescence and 1∶500 for western blotting. Two anti-PCM1 antibodies were used in this study: rabbit anti-PCM1 (A. Merdes, Centre National de la Recherche Scientifique/Pierre Fabre) used at 1∶10,000 for immunofluorescence and rabbit anti-PCM1 (H-262; Santa Cruz Biotechnology, Inc.) used at 1∶100 for immunofluorescence. Mouse anti–polyglutamylated tubulin (GT335; C. Janke, Centre de Recherches de Biochemie Macromoléculaire) was used at 1∶5000, mouse anti–γ-tubulin (GTU-88; Sigma-Aldrich) at 1∶1000 for immunofluorescence, and mouse anti-centrin (clone 20H5; gift from J. Salisbury, Mayo Clinic, Rochester, NY) at 1∶2000 for immunofluorescence. Two GFP antibodies were used in this study: rabbit anti-GFP antibody was generated and used as previously described [35] and rat anti-GFP (GF090R; Nacalai USA, Inc.) was used at 1∶2000 for immunofluorescence. Mouse anti-Myc (9E10; Sigma-Aldrich) was used at 1∶500 for immunofluorescence and 1∶2000 for western blotting. Mouse anti-phosphotyrosine (4G10; Millipore) was used at 1∶1000 for immunofluorescence. Rabbit anti-p38 (C-20; Santa Cruz Biotechnology, Inc.) was used at 1∶5000 for western blotting. Rabbit anti-GAPDH was used at 1∶10,000 for western blotting (G9545; Sigma-Aldrich).

### RNA Interference

The PCM1 shRNA construct has been previously described [18]. PCM1 shRNAs were transfected into HeLa cells using Lipofectamine LTX following the manufacturer’s instructions (Invitrogen). 24 hours post-transfection, HeLa cells were arrested with excess thymidine for 24 hours and released from thymidine for 9 hours to enrich for G_2_ cells.

FOP siRNA #1 oligos have been previously reported [21]. Briefly, FOP siRNA #1 oligos were designed against the following sequence: 5′-gtgatcaggcgctgtcaac-3′ and ordered from Thermo Scientific, duplex ready, 2′-deprotected, desalted, with UU 3′-overhangs. FOP siRNA #2 and #3 oligos targeting 5′-ggtggacccttattattag-3′ and 5′-tcagtgatgttgcggatta-3′, respectively were also ordered from Thermo Scientific. Nontargeting siRNA oligos were used as a control (D-001210-02-05; Thermo Scientific). siRNAs were transfected into HeLa and RPE-1 cells at a final concentration of 50 nM using Lipofectamine RNAiMAX following the manufacturer’s instructions.

### Cell Culture and Transfection

HeLa and RPE-1 cells were cultured in DMEM or DMEM/F12 50/50 medium (Cellgro) +10% fetal bovine serum (Atlanta Biologicals), respectively. ^LAP^BBS4-hTERT-RPE1 cells [26] were provided by M. Nachury (Stanford University, Stanford, CA) and cultured in DMEM/F12 50/50 medium +10% fetal bovine serum (Atlanta Biologicals). Plasmids were transfected using Lipofectamine LTX according to manufacturer’s instructions (Invitrogen). Pools of RPE-1 cells stably expressing pEFP-N1 (RPE-1::GFP) or GFP-resFOP (RPE-1::GFP-resFOP) were produced by transfection followed by selection with 800 µg/ml Geneticin (Invitrogen). For microtubule depolymerization experiments, HeLa cells were released from thymidine-arrest for 9 hours, the last 3 hours incubated with 10 µg/ml nocodazole (US Biologicals), to enrich for G_2_ cells with depolymerized microtubules. For cell cycle arrests, HeLa cells were incubated for 24 hours in DMEM +0.5% fetal bovine serum to arrest in G_1_. For S phase cells, HeLa cells were released from G_1_ by addition of DMEM +10% fetal bovine serum supplemented with 2 mM thymidine (Sigma-Aldrich) and incubated for 18 hours. For G_2_ phase cells, HeLa cells were released from G_1_ by addition of DMEM +10% fetal bovine serum and incubated for 10 hrs, followed by incubation with complete medium supplemented with 10 µg/ml etoposide (Cimprich Lab, Stanford University, Stanford, CA) for 8 hours. Mouse tracheal epithelial cells were cultured as previously described [25], [36], [37].

### Western Blotting and Immunofluorescence

HeLa cells were harvested and lysed in triton buffer (1% triton, 150 mM NaCl, 50 mM Tris pH 8). Insoluble material was pelleted for 5 minutes at 3.3×g and protein concentration determined by Bradford analysis. For immunofluorescence experiments, cells were grown on coverslips coated with poly-L-lysine and fixed with −20°C methanol. Coverslips were washed with PBS and blocked in 3% BSA (Sigma) in PBS +0.1% Triton. Coverslips were incubated in primary antibodies diluted in blocking solution as indicated in “Antibodies” section. Alexa 488- or 594-conjugated secondary antibodies were diluted 1∶500–1∶1000 in blocking solution (Invitrogen). DyLight 649-conjugated secondary antibodies were diluted 1∶200 in blocking solution (Jackson ImmunoResearch). Coverslips of cells were imaged using OpenLab 4.0.4 on an Axiovert 200 M microscope (Carl Zeiss MicroImaging, Inc.) with a Plan-NEOFLUAR 100× (1.3 NA) objective. Images were captured using an Orca-ER cooled CCD camera (Hamamatsu), and were processed using Photoshop (Adobe Systems). Filters of mouse tracheal epithelial cells were imaged using a Leica TCS SP5 AOBS confocal microscope and processed with LAS AF (Leica) and Adobe Photoshop.

### Statistical Analysis

All statistical analyses were conducted with unpaired, two-tailed, Student’s t tests using three independent trials. Values with p<0.05 were considered statistically significant.

## Supporting Information

Figure S1 **FOP costained with centrin.** **(A)** RPE-1 cells stained with antibodies against FOP (green), centrin (red), and PCM-1 (blue). Scale bars: 10 µm; insets: 5× magnification.
(TIF)

Figure S2 **Depletion of FOP antibody reactivity by incubation with purified recombinant protein**. RPE-1 cells stained with antibodies against FOP (green) incubated with purified recombinant FOP or control protein (BSA). Cells are costained with glutamylated-tubulin (red). DNA is stained using DAPI (blue). Scale bars: 10 µm; insets: 5× magnification.
(TIF)

Figure S3 **FOP depletion results in loss of FOP from satellites in G_2_ cells**. HeLa cells transfected with control or FOP siRNA #1 for 48 hours followed by staining with FOP (green) and PCM1 (red). DNA is stained using DAPI (blue). Scale bars: 10 µm; insets: 5× magnification.
(TIF)
