# Supplementary figures and images for: FOP Is a Centriolar Satellite Protein Involved in Ciliogenesis
Source: PLoS One. 2013 Mar 12;8(3):e58589. doi: 10.1371/journal.pone.0058589 (PMC3595297; doi:10.1371/journal.pone.0058589)

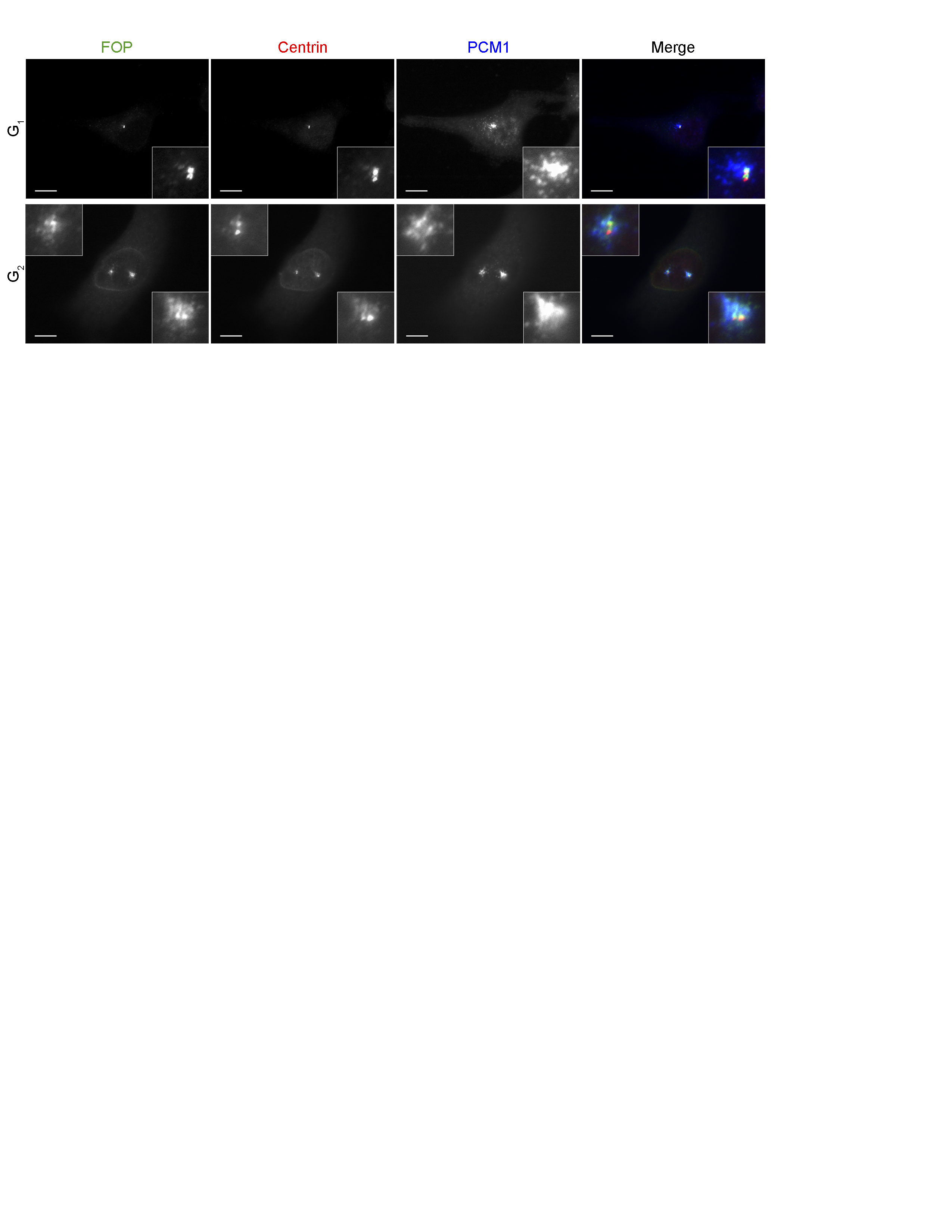

Supplement: Figure S1 — FOP costained with centrin. (A) RPE-1 cells stained with antibodies against FOP (green), centrin (red), and PCM-1 (blue). Scale bars: 10 µm; insets: 5× magnification. (TIF) [file pone.0058589.s001.tif]

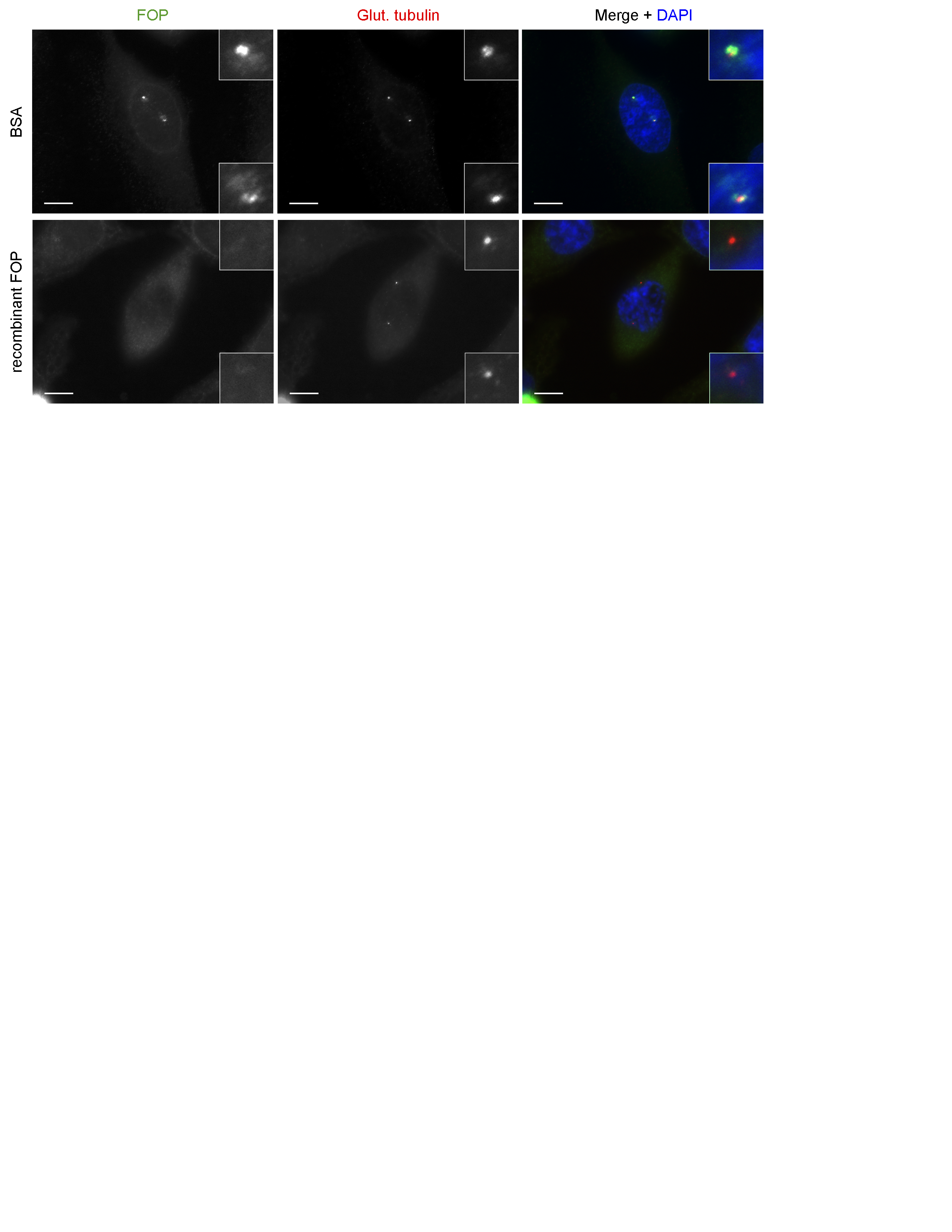

Supplement: Figure S2 — Depletion of FOP antibody reactivity by incubation with purified recombinant protein. RPE-1 cells stained with antibodies against FOP (green) incubated with purified recombinant FOP or control protein (BSA). Cells are costained with glutamylated-tubulin (red). DNA is stained using DAPI (blue). Scale bars: 10 µm; insets: 5× magnification. (TIF) [file pone.0058589.s002.tif]

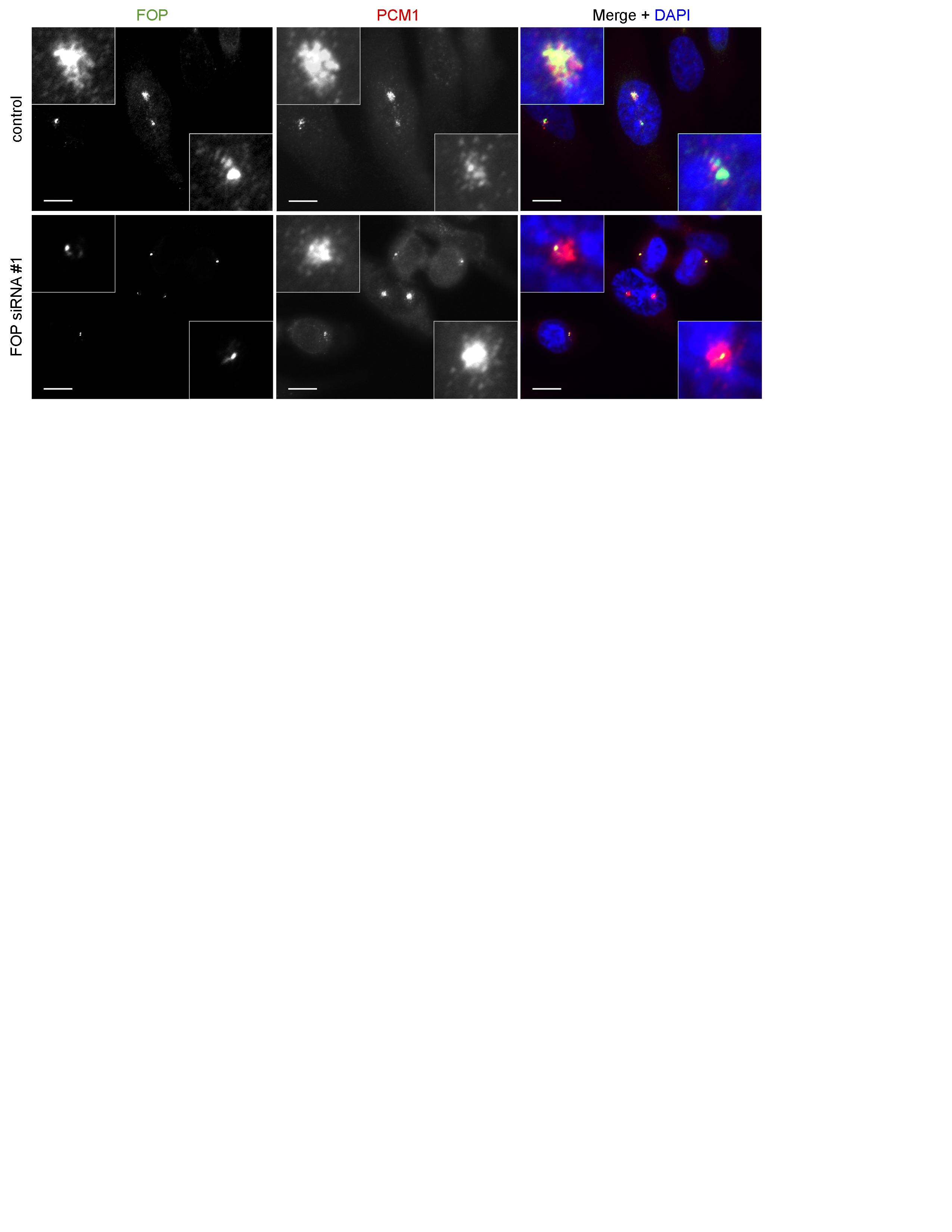

Supplement: Figure S3 — FOP depletion results in loss of FOP from satellites in G2 cells. HeLa cells transfected with control or FOP siRNA #1 for 48 hours followed by staining with FOP (green) and PCM1 (red). DNA is stained using DAPI (blue). Scale bars: 10 µm; insets: 5× magnification. (TIF) [file pone.0058589.s003.tif]
